# Nanomiemgel - A Novel Drug Delivery System for Topical Application - In Vitro and In Vivo Evaluation

**DOI:** 10.1371/journal.pone.0115952

**Published:** 2014-12-29

**Authors:** Jaganmohan Somagoni, Cedar H. A. Boakye, Chandraiah Godugu, Apurva R. Patel, Henrique Antonio Mendonca Faria, Valtencir Zucolotto, Mandip Singh

**Affiliations:** 1 College of Pharmacy and Pharmaceutical Sciences, Florida A&M University, Tallahassee, FL 32307, United States of America; 2 Nanomedicine and Nanotoxicology Group, Physics Institute of São Carlos, USP, 13566-590, São Carlos, SP, Brazil; The University of Tennessee Health Science Center, United States of America

## Abstract

**Aim:**

The objective of this study was to formulate and evaluate a unique matrix mixture (nanomiemgel) of nanomicelle and nanoemulsion containing aceclofenac and capsaicin using in vitro and in vivo analyses and to compare it to a marketed formulation (Aceproxyvon).

**Methods:**

Nanomicelles were prepared using Vitamin E TPGS by solvent evaporation method and nanoemulsion was prepared by high-pressure homogenization method. *In vitro* drug release and human skin permeation studies were performed and analyzed using HPLC. The efficiency of nanomiemgel as a delivery system was investigated using an imiquimod-induced psoriatic like plaque model developed in C57BL/6 mice.

**Results:**

Atomic Force Microscopy images of the samples exhibited a globular morphology with an average diameter of 200, 250 and 220 nm for NMI, NEM and NMG, respectively. Nanomiemgel demonstrated a controlled release drug pattern and induced 2.02 and 1.97-fold more permeation of aceclofenac and capsaicin, respectively than Aceproxyvon through dermatomed human skin. Nanomiemgel also showed 2.94 and 2.09-fold greater Cmax of aceclofenac and capsaicin, respectively than Aceproxyvon in skin microdialysis study in rats. The PASI score, ear thickness and spleen weight of the imiquimod-induced psoriatic-like plaque model were significantly (p<0.05) reduced in NMG treated mice compared to free drug, NEM, NMI & Aceproxyvon.

**Conclusion:**

Using a new combination of two different drug delivery systems (NEM+NMI), the absorption of the combined system (NMG) was found to be better than either of the individual drug delivery systems due to the utilization of the maximum possible paths of absorption available for that particular drug.

## Introduction

Current therapies for the treatment of skin inflammation are not wholly effective and could be attributable to the types of topical delivery systems used. There is therefore a need to develop a controlled-release drug delivery system that would effectively deliver anti-inflammatory agents to reduce pain, inflammation, disease progression and prevent adverse reactions [1], [2]. Aceclofenac is a Non-Steroidal Anti-Inflammatory Drug used in the treatment of inflammation and degenerative disorders of the musculoskeletal system. It is widely prescribed for the treatment of osteoarthritis and rheumatoid arthritis [3], [4], [5]. Capsaicin on the other hand, is used alone or in combination with other anti-inflammatory drugs to effectively reduce itching associated with skin inflammatory conditions [6], [7], [8], [9].

The skin is an exceptionally effective barrier and it prevents the permeation of most of the drugs applied for therapeutic purposes [10], [11]. Very few drugs have the capability to permeate in significant amounts through the skin. Most of the topical dosage forms available on the current market have poor penetration, which leads to poor therapeutic benefit [12], [13]. Hence a delivery system that makes the skin more permeable and penetrates the skin by multiple mechanisms to enhance topical drug delivery is of great formulation interest. Long-term oral administration of aceclofenac causes serious gastrointestinal side effects like GI bleeding and ulceration [14], [15]. Therefore, an improved topical aceclofenac formulation with a high degree of percutaneous permeation could be a useful alternative for the treatment of locally inflamed skin.

One of the most promising drug delivery systems for enhancing skin permeation of drugs is the microemulsion or nanoemulsion system [16], [17]. Nanoemulsions are thermodynamically stable transparent (translucent) dispersions of oil in water stabilized by an interfacial film of surfactant and co-surfactant molecules with droplet size less than 1000 nm. Kakumanu et al. [18] have illustrated in the epidermoid skin carcinoma xenograft mouse model that the nanoemulsion formulation of dacarbazine dramatically increases its efficacy.

**Figure 1:**
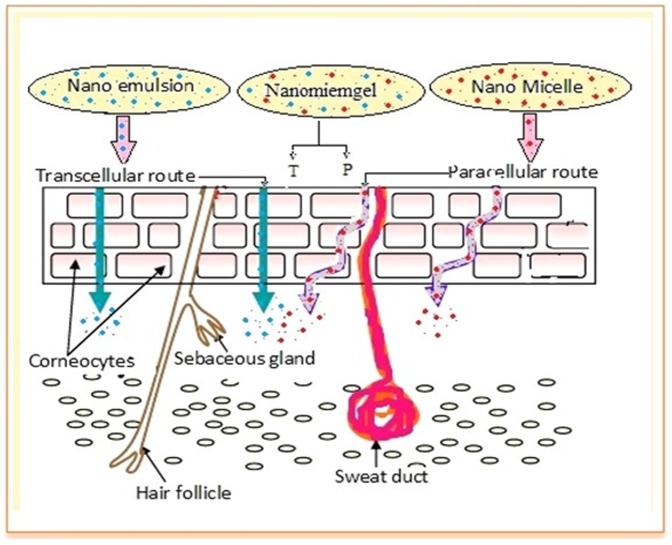
Graphical representation of nanomiemgel and its hypothetical paths of entry into the skin where T: Transcellular & P: Para cellular route of absorption.

In this study, we have developed a drug delivery system called nanomiemgel through a novel formulation strategy, which utilizes the “Multi Absorption Mechanism” (MAM) concept and has a broad applicability. Nanomiemgel consists of two types of matrices; A & B. Matrix A comprises the nanoemulsion whilst matrix B comprises the nanomicelles. The hypothesis of the present study is that every nano drug delivery system is unique and its rate, extent and mechanism of absorption depend on the size, charge and composition of the nano drug delivery system. So, when a combination of completely different drug delivery systems is utilized for the delivery of a drug, the absorption of the combined system would be better than either of the individual drug delivery systems due to the utilization of the maximum possible paths of absorption available for that particular drug (Fig. 1).

The objective of the present research work was to investigate the beneficial effect of the combination of the nanomicelles (NMI) and nanoemulsion (NME) as a novel carrier system for the topical application of aceclofenac (ACE) and capsaicin (CAP). In this study, the *in vitro* permeation of the drug through dermatomed human skin and inflamed mice skin, bioequivalence determination of the nanomiemgel (NMG) with marketed formulation Aceproxyvon (MKT) using dermal microdialysis and the *in vivo* anti-inflammatory activity of the NMG (nanomicelles plus nanoemulsion plus gel) were evaluated.

The plan of work of this research work was to prepare NEM and NMI individually, and incorporation of NEM and NMI at 1∶1 ratio into the gel to get a uniform matrix of the combined drug delivery system (NMG) and then characterization of NEM, NMI and NMG by different means followed by a systematic investigation of these formulation for *in vitro* and *in vivo* efficacy in psoriatic like inflammation model and also to compare the effects with MKT using dermal microdialysis.

## Materials

Polyvinyl alcohol (PVA), dichloromethane, Tween 80, sodium tripolyphosphate (TPP), polyethylene glycol 400 (PEG-400), phosphate buffer saline sachets (PBS, pH 7.4), and trifluoroacetic acid (TFA) were purchased from Sigma Aldrich Co (St Louis, MO). HPLC grade of acetonitrile, water and ethanol were purchased from Sigma Aldrich Co (St Louis, MO).

Aceclofenac (ACE) was purchased from AK scientific mfg corp. (Gardena, CA). Capsaicin (CAP) and Imiquimod (IMQ) were purchased from VWR International (Suwanee, GA). Carbopol (Carbopol 2020 NF) was generously gifted by Lubrizol Advanced Materials, Inc. (Cleveland, OH). Olive oil and Vitamin E TPGS were purchased from VWR Ltd. Aceproxyvon gel (ACE 1.5%, CAP: 0.01%) was purchased from Wockhardt Pharmaceuticals Limited (Mumabai, India). IL-23 antibody and ABC staining immunohistochemistry kit were purchased from Santa Cruz Biotechnology Inc (Santa Cruz, CA).

## Methods

### Formulation of aceclofenac and capsaicin nanoemulsion

The aceclofenac (ACE) and capsaicin (CAP) nanoemulsion (NEM) was prepared by first dissolving 750 mg of ACE and 5 mg of CAP in 8 mL of olive oil and miglyol (1∶1), followed by the addition of 6 mL of “Polysorbate 80 and Transcutol” mixture (1∶1). The oil and surfactant mixture-containing drug were sonicated for about 15 minutes to get clear oil & surfactant mixture. To the mixture, 11 mL of deionized water was added while homogenizing to get a primary emulsion. The obtained o/w emulsion was homogenized further for 5 minutes at 3000 rpm to obtain a microemulsion. This microemulsion when passed through a NanoDEBEE (Bee International, South Easton, MA, USA) at 24,000 psi for 5 cycles resulted in a NEM.

For the measurement of the mean droplet size and polydispersity index (particle size distribution) and ζ-potential, a zeta sizer (Nicomp 380 ZLS (Particle Sizing Systems, Port Richey, FL) was used.

### Formulation of aceclofenac and capsaicin nanomicelle

The ACE and CAP nanomicelle was prepared with Vitamin E TPGS using the solvent evaporation method where the organic solvent was removed through evaporation. The Vitamin E TPGS (7.55 gm), aceclofenac (750 mg) and capsaicin (5 mg) were added to 2 mL of acetone. When a clear solution was obtained, 25 mL of distilled water was added. Then the organic solvent was removed gradually through evaporation. Change of solvent quality and hence, selectivity, from organic to aqueous was gradual; the polymer and the drugs were able to aggregate into micelles rather than precipitating from the solution into the bulk. The solvent of choice was acetone, due to its high water miscibility and low vapor pressure, which simplified the solvent removal.

### Preparation of nanomiemgel

50 mL of purified distilled water and propylene glycol (1∶1) were put into a beaker and heated up to 70°C. EDTA (0.5%) and pluronic F-127 (0.5%) were added into the warmed purified water with continuous stirring after which the mixture was cooled down to 50°C. The mixture was then added to carbopol (1 g) under continuous low rpm stirring to form a uniform gel that was free from lumps and bubbles. The pH of gel was then neutralized with triethanolamine (TEA). After cooling the gel phase to 40°C, the NEM and NMI formulations were incorporated into the carbopol gel and mixed uniformly to obtain the NMG. The NEM and NMI were dispersed into the carbopol gel to achieve the final concentration of ACE and CAP at 1.5% & 0.01% respectively, whereas the final concentration of carbopol was maintained at 2%. This NMG was used for the drug release, skin permeation and other *in vivo* studies.

### Texture analysis

Different gel formulations were placed one after other on a cone shaped plate and subjected to texture analysis with the TA.XT Plus texture analyzer (Stable Micro Systems, Surrey, UK) interfaced with a computer. The principle of operation is that when a 10 g surface trigger is attained (i.e. the point at which the disc's lower surface is in full contact with the product), the disc proceeds to penetrate to a depth of 30 mm. At this point (most likely to be the maximum force), the probe returns to its original position. The 'peak' or maximum force is considered as a measurement of firmness i.e. the higher the value, the firmer the sample is. The area of the curve up to this point is taken as a measurement of consistency i.e. the more the value, the thicker the consistency of the sample.

**Figure 2:**
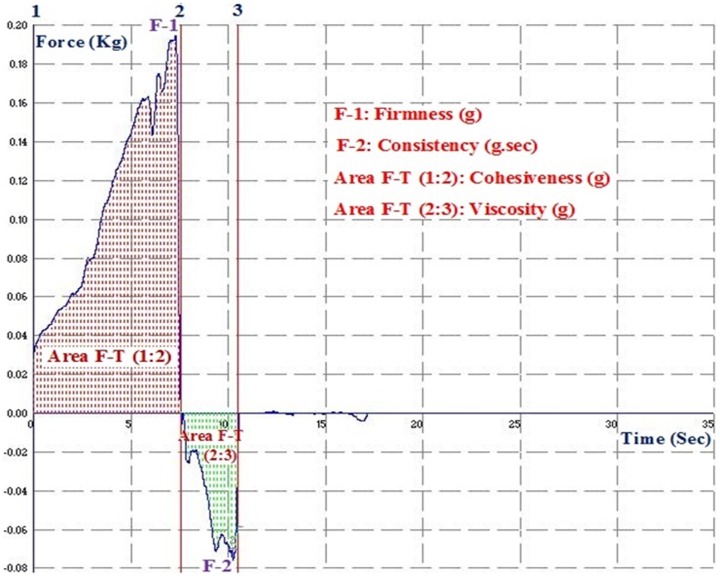
Typical graph representing the texture analysis of a gel.

The negative region of the graph, produced on probe return, is as a result of the weight of the sample which is lifted primarily on the upper surface of the disc on return(due to back extrusion) and hence gives again an indication of consistency/resistance to flow off the disc. The maximum negative force is taken as an indication of the cohesiveness of the sample i.e. the more negative the value, the more 'cohesive' the sample. The area of the negative region of the curve may be referred to as the 'work of cohesion' i.e. the higher the value, the more resistant the sample is to withdrawal which is an indication of the cohesiveness and also the consistency/viscosity (Fig. 2).

All the measurements were made on gels equilibrated to ambient temperature. The gels were compressed twice at 0.5 mm/s speed until 10–15 percent of their original amount was left. The results were reported as the means of triplicate tests.

### Atomic Force Microscopy

Topographical and phase images were obtained for all the nanoformulations prepared. In order to determine the size of the NEM, NMI and NMG and to obtain high resolution images, Atomic Force Microscopy (AFM) analyses were carried out using a Nanosurf Flexa microscope, Switzerland, in the non-contact dynamic mode. The NEM and NMI suspensions were first diluted into ultra-pure water (18 MΩ.cm^−1^) at 1% v/v, whereas the NMG suspension was diluted into ultrapure water at 0.1% v/v. The diluted suspensions were then mixed on a vortex for 1 minute. Subsequently, 5 µL of the diluted nanoformulations were poured on optical microscope slides (5 mm ×5 mm) previously cleaned upon sonication in ethanol. The slides containing the drop casting samples were dried at room temperature (22°C) in a desiccator (silica gel) for 3 days. All measurements were performed at room temperature (22°C) under controlled humidity (20–30%). The Gwyddion software was employed for images treatment for quality enhancement.

### HPLC analysis

**Figure 3:**
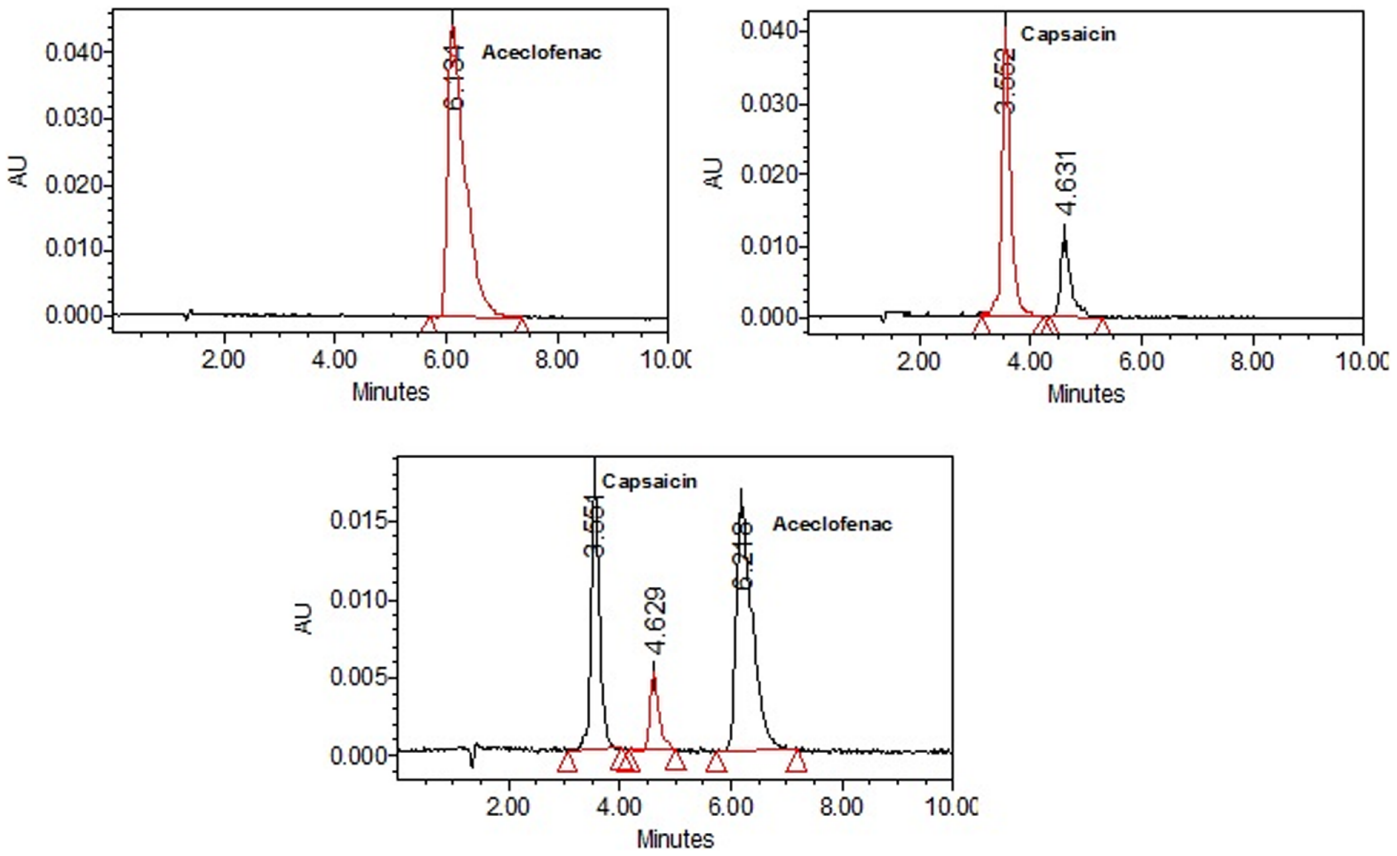
Chromatograms showing separation of aceclofenac and capsaicin. Mobile Phase: Acetonitrile+10 mM acetate buffer (55+45 v/v) pH adjusted to 3 with acetic acid. Flow: 1 mL/min. Column: Symmetry C18 column, 150×4.5 mm.

HPLC system (Waters Corp, Milford, MA) with a symmetrical reverse phase C18 analytical column (5 mm, 4.6×250 mm) was used for the analysis of ACE and CAP present in the NMG. The mobile phase used was 10 mM acetate buffer (pH adjusted to 3) and acetonitrile at 45∶55 v/v with a flow rate of 1 mL/min. Retention time for ACE and CAP were 6 and 3.5 min respectively (Fig. 3). All the samples were analyzed at 275 nm.

### *In-vitro* drug release

*In vitro* drug release study was performed using USP type II apparatus in 500 ml of phosphate buffer pH 6.8, with speed and temperature maintained at 100 rpm and 37±0.5°C, respectively. 2 gm of each formulation (NMG, NMI, NEM and MKT) and free drug gel (FD) (equivalent to 30 mg ACE and 0.2 mg CAP) was placed in a dialysis bag made up of cellulose (molecular weight cut off of 12,000 g/mole; Sigma, USA) and subjected to the dissolution study. 1 mL samples were withdrawn at regular time intervals (1, 2, 4, 6, 8, 12, 24, 48 and 72 h) and same amount of dissolution medium was replaced after each withdrawal. The withdrawn samples were analyzed for the drug content by using developed RP-HPLC method at 275 nm.

### Stability studies

Different formulations (NMG, NMI and NEM) including MKT were filled in the glass vials and subjected to the stability studies as per the ICH guidelines at 2–8°C (refrigeration condition), 25±2°C/60±5%RH (room temp) and 40±2°C/75±5%RH (accelerated condition) for 6 months. Appearance and clarity were analyzed by visual inspection. The particle size of NMG and drug content were evaluated using zeta sizer and HPLC respectively.

### Ex vivo permeation through dermatomed human skin

Dermatomed human skin was obtained from Allosource (Centennial, CO) with a thickness of 0.5–0.1 mm. The skin was then stored at −80°C until used. The dermatomed human skin was thawed and washed with distilled water for 30 min to remove the excess of glycerol. Skin permeation studies were performed using established procedures. The human skin permeation studies were performed by mounting the dermatomed human skin on Franz diffusion cells (Permegear Inc., Riegelsville, PA). The surface area of the dermatomed human skin exposed to the formulation in the donor chamber was 0.64 cm^2^ and the receiver fluid volume was 5 ml.

Free drug (FD), Aceproxyvon (MKT), NEM, NMI and NMG were applied evenly on the surface of the human skin in the donor compartment. The skin permeation study was performed using six diffusion cells and represented as an average of six cells. The receiver compartment was filled with 1% w/v Tween 80 in PBS (pH 7.4) and stirred at 300 rpm. The temperature of the receiver compartment was maintained at 37±0.5°C using a circulating water bath to simulate the skin temperature at physiological level. To replicate the clinical conditions, a non-occlusive condition was followed and the surface of the skin was exposed to the surrounding air. After 24 h of skin permeation, the receiver fluid was collected and centrifuged at 15000 rpm for 15 min and analyzed for drug content using the HPLC method.

### Skin extraction

For the evaluation of drug retention in dermatomed human skin, the entire dosing area (0.64 cm2) was collected with a biopsy punch. Stratum corneum (sc), epidermis (ed) and dermis (de) were separated using cryotome. SC, epidermis and dermis were minced and boiled with 250 µL PBS (pH 7.4) separately for 10 min. To these samples 250 µL of acetonitrile was added to solubilize the drug. All the samples were then centrifuged at 15000 rpm for 15 min. The supernatant was collected and analyzed by HPLC for drug content.

### Visualization of skin penetration

Microscopic analysis on the rat skin was carried out following skin permeation study with FITC (flourescein isothiocyanate) dye loaded NEM, NMI and NMG to demonstrate the superior skin permeation ability of NMG over NEM and NMI using confocal laser scanning microscopy (Leica Microsystems Inc., IL, USA). Cryosectioning was done to get both cross and lateral sections up to 400 µm depth using cryotome (Shandon, UK). The cryotomed skin sections of NEM, NMI and NMG were visualized and analyzed for skin associated fluorescence using a 10× objective piece throughout the study for all the samples.

### Comparison of dermal drug delivery of NMG and aceproxyvon using microdialysis

#### *In Vitro* Evaluation of Probe Recovery

The transfer rate of the probes, possible binding effect and *in vitro* recovery of ACE and CAP were assessed as per previously described procedures [19]. The standard stock solution of ACE (10 mg/ml) and CAP (0.01 mg/ml) was prepared in 1% (w/v) Tween-80 containing PBS solution. A linear microdialysis (MD) probe with 10 mm dialysis membrane (LM-10, Bioanalytical Systems, West Lafeyetter, IN, USA) was used for these studies.

#### *In Vivo* Evaluation of Probe Recovery

*In vivo* recovery of ACE and CAP was done by retrodialysis methods. CD SD hrBi hairless rats were anaesthetized with an intraperitoneal injection of urethane (1.5 g/kg) and placed on a temperature controlled heating pad (37±2°C). To do the dermal implantation of the LM-10 probe, the skin of the dorsal region of the rat was punctured with 19-gauge intravenous needle (BD Company, Franklin Lakes, NJ, USA) and MD probe was inserted through the guiding needle cannula. Then the needle was retracted leaving the dialysis membrane in the skin horizontally. ACE and CAP standard stock with 1% (w/v) Tween-80 in Krebs-Ringer solution was passed through the probe using an infusion pump at a flow rate of 2 µl/mL and the dialysate samples were collected every 60 min for 480 min. The recovery was determined from the ratio of the concentration loss to the initial concentration in the perfusate. 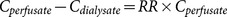



#### *In vivo Skin* Microdialysis

*In vivo Skin* microdialysis was done using the MD setup with slight modification of standard procedure [20]. In short, the linear MD probes were used and were continuously perfused with 1% (w/v) Tween-80 in Phosphate Buffer Saline (PBS) solution at a flow rate of 2 µl/min. Dialysate samples were collected every hour into micro-fraction collector throughout the study period. Two MD probes were implanted in parallel position on the dorsal surface of the rat skin. The needle was inserted into the skin very carefully so that the needle could be visible through the superficial skin layer. For the topical application of different formulation gels, the donor chambers were fixed on the rat skin at the point where MD membranes were implanted using glue. 200 µl of FD, MKT, NEM, NMI and NMG gels (n = 4) were applied on the skin surface using donor chambers. Subsequently, the dialysate samples were collected every hour for 24 h and were analyzed for the ACE & CAP levels using HPLC.

The concentrations of ACE and CAP at different time points in the dialysate samples were subjected to pharmacokinetic analysis to estimate various pharmacokinetic parameters like maximum plasma concentration (C_max_), time to reach maximum concentration (t_max_), area under the plasma concentration-time curve (AUC_0→t_ and AUC_0→ω_) and elimination half life (t_1/2_) using WinNonlin software.

### *In vivo* inflammatory model

#### Animals

C57BL/6 mice (6 weeks old; Charles River Laboratories, Wilmington, MA) were grouped and housed (n = 5 per cage) in cages with Tek-Fresh bedding. The animals were kept under controlled conditions of 12∶12 h light: dark cycle, 22±2°C and 50±15% RH. The mice were fed with Harlan (Teklad) and water ad libitum. The animals were housed at Florida A & M University in accordance with the standards of the Guide for the Care and Use of Laboratory Animals and the Association for Assessment and Accreditation of Laboratory Animal Care (AAALAC). The animals were acclimatized to laboratory conditions for one week prior to all experiments. The protocol of the animal study was approved by the Institutional Animal Care and Use Committee (IACUC), Florida A&M University, FL.

#### Imiquimod (IMQ) induced psoriatic plaque like model

The model selected was IMQ-induced psoriatic-like plaque in mouse skin because it closely resembles the clinical presentation of human psoriasis with respect to the erythema, skin thickening, scaling, epidermal alterations, neoangiogenesis and infiltration of inflammatory proteins such as T cells, neutrophils and dendritic cells [21], [22].

C57BL/6 mice of age 8–11 weeks were kept under specific pathogen-free conditions. Topical application of IMQ suspension was carried out for 5 consecutive days on the shaved backs of the mice. The dose of IMQ applied (5 mg/day) was optimized based on the induction of skin inflammation. Subsequently, the inflamed skin area was treated with FD, MKT, NEM, NMI and NMG gels topically for 5 consecutive days. Groups subjected to no IMQ and IMQ only treatments were utilized as the negative (NC) and positive (PC) control groups, respectively.

#### Scoring severity of skin inflammation, ear thickness and spleen enlargement

An objective scoring system was developed based on the clinical Psoriasis Area and Severity Index (PASI) to score the severity of inflammation induced on the back of the mice. The extent of erythema, scaling and thickening was scored independently on a scale from 0 to 4: 0, none; 1, slight; 2, moderate; 3, marked; 4, very marked. The scoring was performed every 24 h for 5 days.

The reduction in the thickness of the inflamed ears of the mice was measured using caliper (Marathon Caliper, Digital Electronic, VWR, USA) and this parameter was also used to assess the treatment success of the inflammation induced after the topical application of different gel formulations. After 5 days of treatment with the different formulations, the mice were sacrificed and the spleen weights were taken to determine the differences among the different groups and to evaluate the efficiency of different formulations.

#### Histology

The inflamed skin of mice was collected at the end of the experiment and stored in 10% neutral phosphate buffered formalin. Following fixation, the samples were dehydrated and embedded in paraffin. Microtome sections of about 5 mm thickness were taken from the inflamed skin and stained with hematoxylin as well as eosin. The Olympus BX40 light microscope equipped with computer-controlled digital camera (DP71, Olympus Center Valley, PA) was used to visualize the images on the slides.

#### Immunohistochemistry (IHC)

IL-23 is a cytokine produced by macrophages that takes an important role in the inflammatory response [23], [24]. Hence, IHC study of IL-23 was performed as per the procedure described by Chougule et al. [25]. In brief, formalin-fixed, paraffin-embedded skin sections were used for IHC studies according to the protocol specified in the ImmunoCruz mouse ABC staining kit (SantaCruz Biotechnology Inc, CA).

The section slides were washed in xylene and hydrated with different concentrations of alcohol. The slides were incubated with the primary antibody against IL-23 overnight at 4°C. Horseradish peroxidase-conjugated secondary antibody was applied to locate the primary antibody. The specimens were stained with DAB chromogen and counterstained with hematoxylin. The presence of brown staining was considered as a positive identification for activated IL-23. The Olympus BX40 light microscope equipped with computer-controlled digital camera (DP71, Olympus Center Valley, PA) was used to visualize the images on the slides.

#### Permeation through inflamed mice skin

The psoriatic dorsal skin portions of the mice following treatment with the different formulations were subsequently collected and subjected to drug permeation studies using a similar experimental set up as for the permeation studies with dermatomed human skin. The different mounted skin portions were all then treated with NMG. The concentrations of ACE and CAP were maintained as same as used for the permeation studies through the dermatomed human skin.

The permeation study was conducted to ascertain the effect of inflammation (plaque, scaling and epidermal thickness) on the skin permeation of drugs to determine the efficiency of the topical delivery of these formulations during psoriasis treatment.

### Combination Index

The combination index (CI) value was calculated separately for different parameters like the cumulative percent of drug permeated into the receptor compartment, amount of drug retained in the different layers of the skin, the thickness of the inflamed ear of the mice treated with IMQ to induce psoriasis and the PASI score to evaluate the combined effect of NEM and NMI. The CI was calculated using following equation [26]: 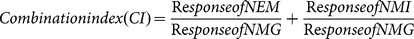



The CI values were interpreted as follows: CI>1.3: antagonism, CI = 1.1 to 1.3: moderate antagonism, CI = 0.9 to 1.1: additive effect, CI = 0.8 to 0.9: slight synergism, CI = 0.6 to 0.8 moderate synergism, CI = 0.4 to 0.6: synergism, CI = 0.2 to 0.4: strong synergism.

### Statistical analysis

The ACE and CAP content of the skin tissue was expressed as mg per g of the tissue. The differences in the skin permeation among the formulations FD, MKT, NEM, NMI and NMG gels were examined using ANOVA. The means were compared between two groups using the student's t test. Mean differences with p<0.001 were considered to be significant. All data is expressed as the mean ± S.D., n = 3.

## Results

### Physical characterization of NMG

The NEM and NMI, each containing ACE and CAP had mean particle size of 229±16 nm and 185±10 nm, respectively and polydispersity index (PI) of 0.18±0.06 and 0.12±0.08 respectively. The entrapment efficiency of ACE and CAP in NEM was 96.74±4.24% and 95.72±3.58%, respectively whereas for NMI, was 94.88±3.76% and 92.69±3.08%, respectively.

### Texture Analysis

The firmness, consistency, cohesiveness and viscosity were determined to optimize the texture parameters of the different formulations. The different texture parameters of the nano formulations, FD and MKT gels were given in Table 1. All the four parameters were found to be least for FD compared to the other formulations because there were no other formulation components except the drug in the blank carbopol gel. MKT gel exhibited higher firmness (294.61±12.55 g) and consistency values (1402.70±53.25) but lesser cohesiveness (−41.12±4.05) whereas moderate firmness (184.84±8.07), consistency (791.10±30.09) and higher cohesiveness (76.11±4.11) values were observed for NMG.

**Table 1:** Various rheological measurements of different formulations after performing the texture analysis.

Formulation	Firmness (g)	Consistency (g.sec)	Cohesiveness (g)	Index of Viscosity (g.sec)
	Force 1	Area F-T 1∶2	Force 2	Area F-T 2∶3
FD	85.58±4.37	210.51±9.12	−27.66±2.45	−50.91±3.56
MKT	294.61±12.55	1402.70±53.25	−41.12±4.05	−158.45±7.23
NEM	204.83±7.51	804.06±29.76	−48.23±3.11	−99.96±5.14
NMI	158.85±6.44	731.26±34.61	−108.09±5.07	−201.02±10.15
NMG	184.84±8.07	791.10±30.09	−76.11±4.11	−148.43±9.88

Data expressed as mean±SD (n = 6).

### *In vitro* drug release

**Figure 4:**
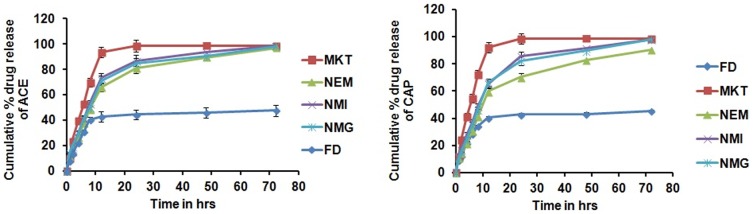
*In vitro* drug release of a) Aceclofenac b) Capsaicin from different formulations in pH 6.8 phosphate buffer. Data expressed as mean±SD (n = 6). Cumulative % of ACE & CAP released from FD after 24 h was significantly less when compared to NMG, NMI, NEM and MKT. Significant where *p<0.05.

The release of ACE and CAP from NMG was determined to be an intermediate between NMI and NEM. The pattern of drug release of ACE and CAP was almost similar for NEM, NMI and NMG and followed first order release kinetics with a best fit R^2^ value of less than 0.99. No sustained release of drug was determined for FD whilst there was sustained drug release for only 10 hr for MKT. However, for NEM, NMI and NMG, the drug was released in a sustained manner for more than 72 hr (Fig. 4).

### Atomic Force Microscopy

**Figure 5:**
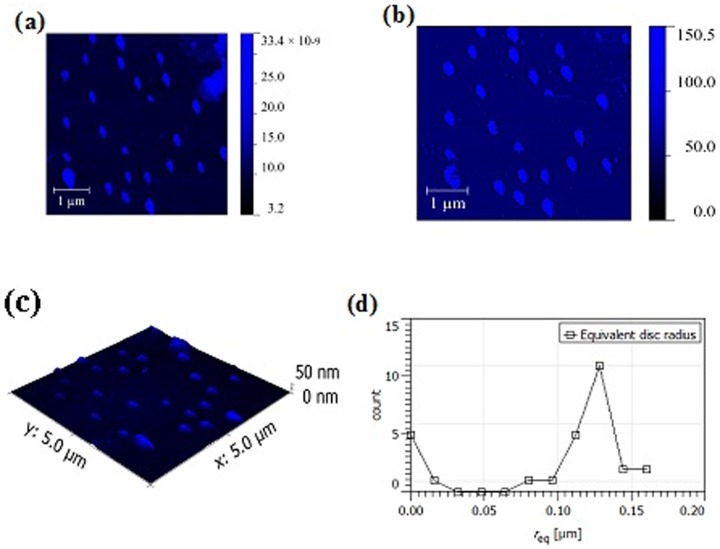
AFM Images for NEM formulation. (a) Topographic (b) Phase mode (c) 3D topography (d) Mean NP radius.

**Figure 6:**
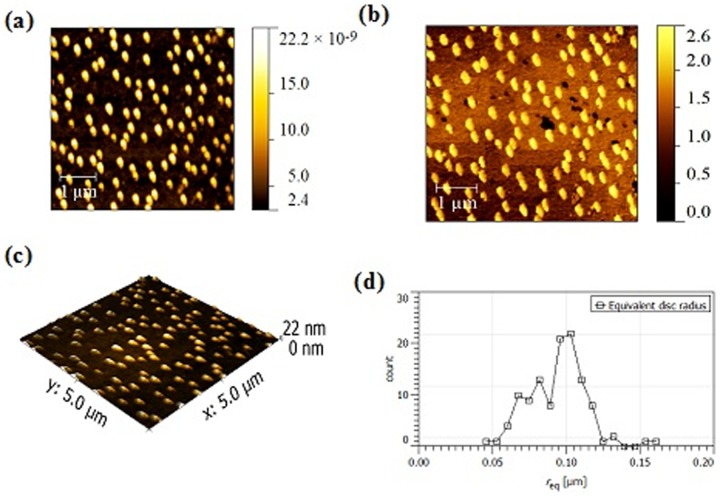
AFM Images for NMI formulation. (a) Topographic (b) Phase mode (c) 3D topography (d) Mean NP radius.

**Figure 7:**
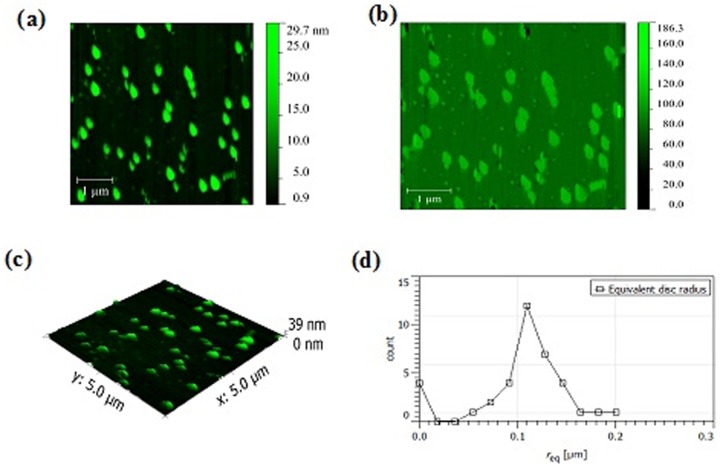
AFM Images for NMG formulation. (a) Topographic (b) Phase mode (c) 3D topography (d) Mean NP radius.

Topographic, phase mode images and mean nanoparticle radius of NEM, NMI and NMG are shown in Figs. 5–7. The dilution/drop-casting samples preparation method allowed the visualization of individual nanoparticles without aggregation even for NMG samples. All samples exhibited a globular morphology with an average diameter of 200, 250 and 220 nm for NMI, NEM and NMG, respectively.

### Stability studies

**Figure 8:**
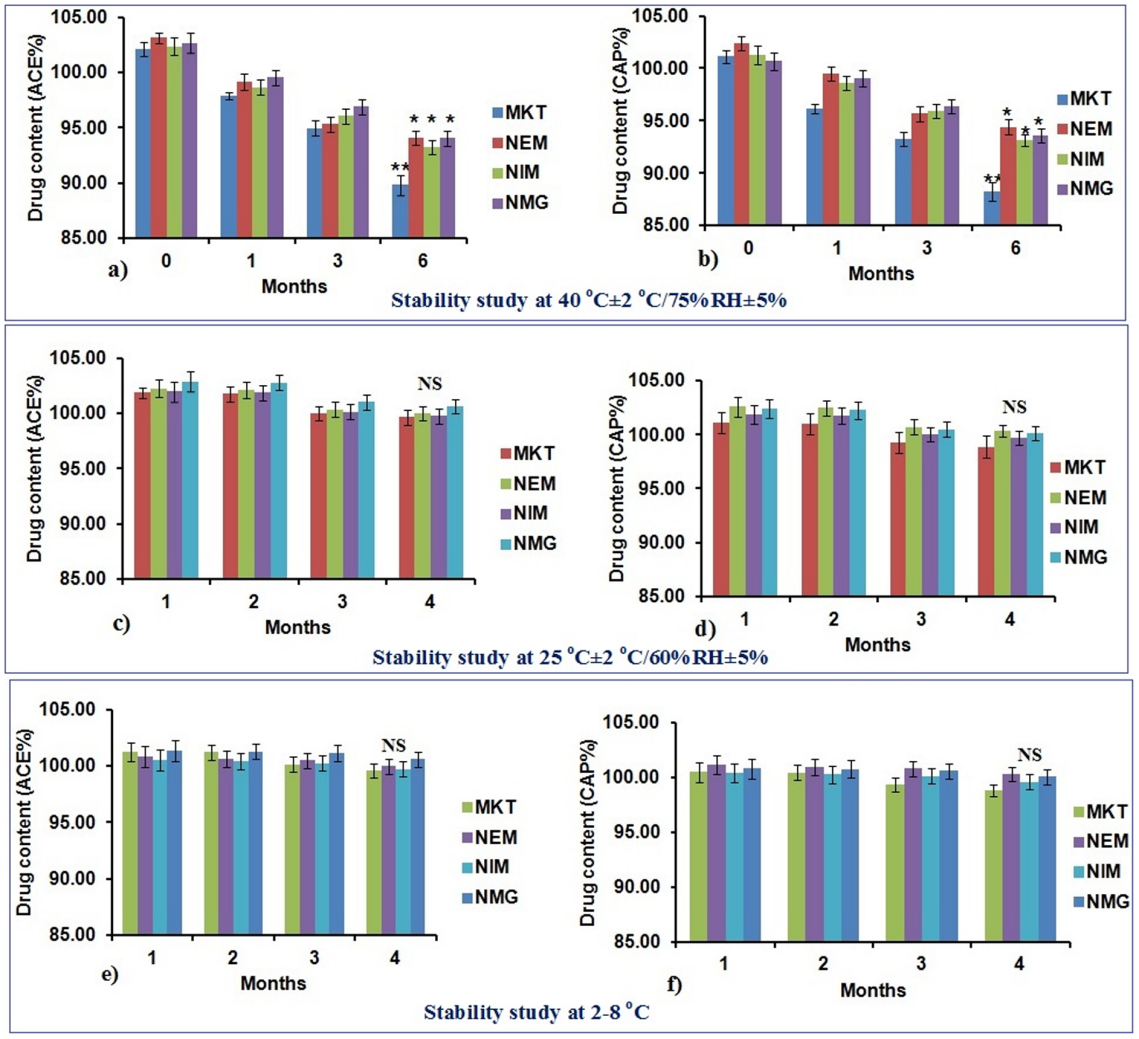
Stability study results of a) & b) are ACE and CAP at 40°C±2°C/75%RH±5%RH, c) & d) are ACE and CAP at 25°C±2°C/60%RH±5%RH, e) & f) are ACE and CAP at 2–8°C. There was a significant decrease in drug content at 6^th^ month compared to 0 month in the formulations stored at 40°C but not in the samples stored at 25°C and 2–8°C and the decrease was more significant with marketed formulation. Data represent mean±SD, n = 6, significant where *p<0.05, **p<0.001, NS: Not significant compared to % drug content in first month.

The particle size of MKT, NEM, NMI and NMG remained unchanged when stored at refrigeration temperature and room temperature. However, there was an increase in the particle size and a decrease in drug content for the different formulations when they were stored at accelerated conditions (40±2°C/75±5% RH) for 6 months. The average particle size for the nano formulations increased from the initial size from 0 to 6 months as follows; NEM: 192±13 to 241±24 nm, NMI: 65±11 to 102±23 nm and NMG: 139±18 to 185±26 nm. The changes in particle size at 25±2°C/60±5% RH were; for NEM: 192±13 to 208±11 nm, NMI: 65±11 to 81±14 nm and NMG: 139±18 to 156±16 nm and the changes at refrigeration temperature (2–8°C) were; for NEM: 192±13 to 201±10 nm, NMI: 65±11 to 77±12 nm and NMG: 139±18 to 145±14 nm. Similarly, there was decreased drug content observed for both ACE and CAP for all the formulations; however, the decrease was more prominent for MKT than the nano formulations (Fig. 8).

### Ex vivo skin permeation through dermatomed human skin

**Figure 9:**
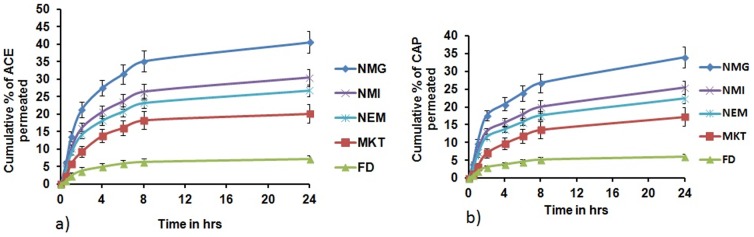
Permeation of aceclofenac and capsaicin through dermatomed human skin. a) Permeation of aceclofenac b) Permeation of capsaicin. Cumulative % of ACE & CAP permeated after 24 h through dermatomed human skin from NMG was significantly more compared to FD, MKT, NEM and NMI. Data represent mean±SD, n = 6, Significant where *p<0.05.

The permeation of ACE and CAP into the receiver compartment is illustrated in (Fig. 9). The cumulative amounts of ACE and CAP permeating into the receiver compartment from NMG after 24 hr were 5.68 and 5.63-fold more than FD and 2.02 and 1.97 fold more than MKT, respectively (Table 2).

**Table 2:** Percentage of drug permeated through dermatomed human skin and retention of drug (µg/gm) in different layers of the human skin *in vitro*.

Percentage of drug permeated & retention of drug (µg/gm) in different layers of the skin								
Formulation	% of drug permeated		Stratum Corneum (SC)		Epidermis (ED)		Dermis (DE)	
	ACE	CAP	ACE	CAP	ACE	CAP	ACE	CAP
FD	7.15±0.91	6.03±0.77	502.08±23.08	3.15±0.43	199.28±23.19	1.79±0.23	159.22±19.21	1.01±0.20
MKT	20.11±2.39	17.27±1.89	1440.50±62.93	8.84±0.22	783.54±37.48	4.29±0.29	523.86±22.91	2.59±0.15
NEM	26.78±1.39	22.41±1.29	1766.83±76.81	11.19±0.47	1270.19±34.14	5.57±0.24	722.84±31.62	3.21±0.15
NMI	30.43±2.04	25.45±1.89	1965.91±85.47	12.45±0.53	1413.31±37.99	6.27±0.27	806.65±35.28	3.58±0.17
NMG	40.58±3.15	33.94±2.92	2488.53±108.19	15.77±0.66	1789.26±48.08	7.85±0.34	1047.63±45.82	4.65±0.22
NMG/FD	5.68	5.63	4.96	5.01	8.98	4.39	6.58	4.60
NMG/MKT	2.02	1.97	1.73	1.78	2.28	1.83	2.00	1.80

Data expressed as mean±SD (n = 6), significant where *p<0.05.

**Figure 10:**
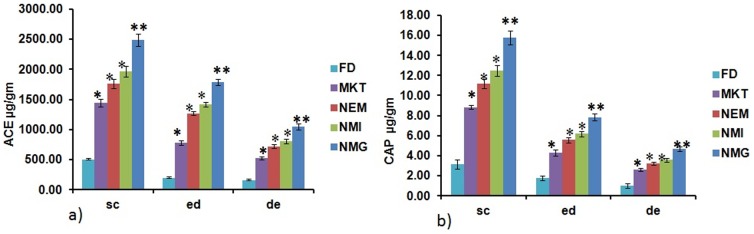
Retention of total aceclofenac & capsaicin in stratum corneum (sc), epidermis (ed) & dermis (de) of dermatomed human skin after 24 h. a) Retention of aceclofenac b) Retention of capsaicin. Data represent mean±SD, n = 6, significant where *p<0.05, **p<0.001 vs FD or positive control.

The SC, epidermal and dermal retention of ACE and CAP after 24 hrs for the different formulations are shown in (Fig. 10). The amounts of ACE and CAP retained in the SC for NMG after 24 hr was 4.96 and 5.01-fold more than FD and 1.78 and 2.28-fold more than MKT, respectively. The retention of ACE and CAP after 24 hr was also determined to be significantly more for NMG than both the FD and MKT.

The retention of ACE and CAP in the epidermis for NMG after 24 hr was 8.98 and 4.39-fold more than FD and 2.28 and 1.83-fold more than MKT respectively, where as the retention of ACE and CAP in the dermis for NMG after 24 hr was 6.58 and 4.60 fold more than FD and 2.0 and 1.80-fold more than MKT, respectively (Table 2).

### Visualization of skin penetration

**Figure 11:**
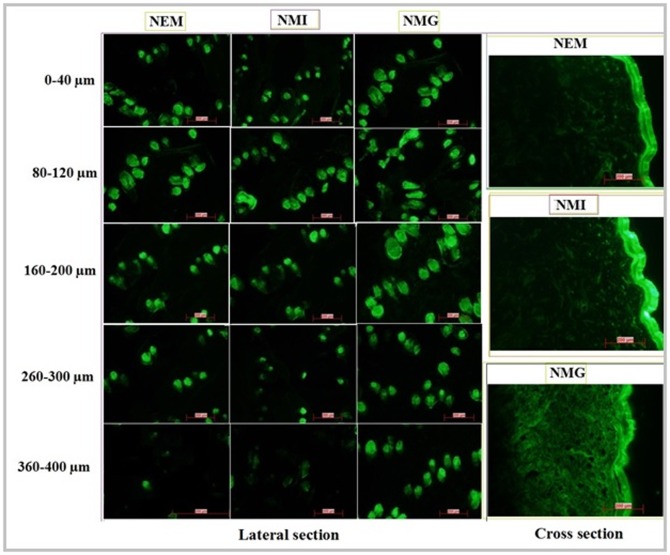
Study of in vitro skin permeation of FITC loaded NEM, NMI and NMG across the rat skin using confocal laser scanning microscopy.

Results from the confocal laser scanning microscopy confirmed that FITC loaded NMG was able to penetrate more and deeper into different skin layers compared to FITC loaded NEM and NMI (Fig. 11). Microscopic studies also demonstrated that there was a decreased green fluorescence signal from FITC with increased depth in cryotomed skin sections with NEM and NMI where as with NMG significantly intense green fluorescence signal was observed especially in skin sections correspond to 260–400 µm depth.

### In vitro/in vivo microdialysis recovery studies

The *in vitro* recovery of ACE and CAP estimated from the bulk solution was 29±6% (*r*2 *less than* 0.99) and 26±4% (*r*2 *less than* 0.99). The plot of “Dialysate” versus “bulk” concentrations exhibited a linear relationship at the concentrations tested.

**Figure 12:**
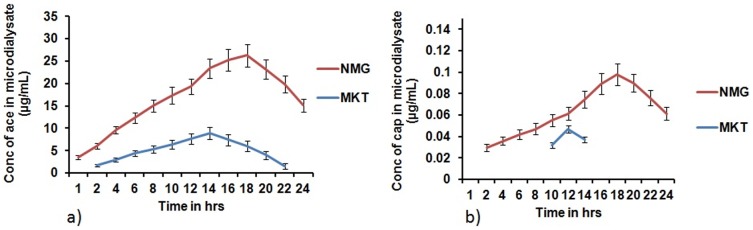
Mean protein-unbound drug concentration–time. Profile of a) aceclofenac and b) capsaicin in microdialysate samples after topical application of nanomiemgel (NMG) and marketed gel (MKT) in hairless rats. Cmax of both ACE and CAP obtained in microdialysate samples from NMG were significantly more than the MKT. Data represent mean±SD, n = 3, Significant where *p<0.05.

ACE and CAP were both detectable in the dialysates of NMG for 24 hrs, whereas in the case of MKT, ACE was not detectable at the 24 hr time point and CAP was detectable at only three time points i.e. 10, 12 and 14 hr. The respective C_max_ of ACE and CAP for NMG (26.24 µg/mL and 0.098 µg/mL) were 2.94 and 2.09-fold more than MKT (8.94 µg/mL and 0.047 µg/mL) (Fig. 12) which signifies the supra bioavailability of NMG. The t_max_ of both ACE and CAP for NMG were 18 hr whereas for MKT, the t_max_ for ACE and CAP were 14 hr and 12 hr respectively.

### Imiquimod (IMQ) induced psoriatic plaque like model

**Figure 13:**
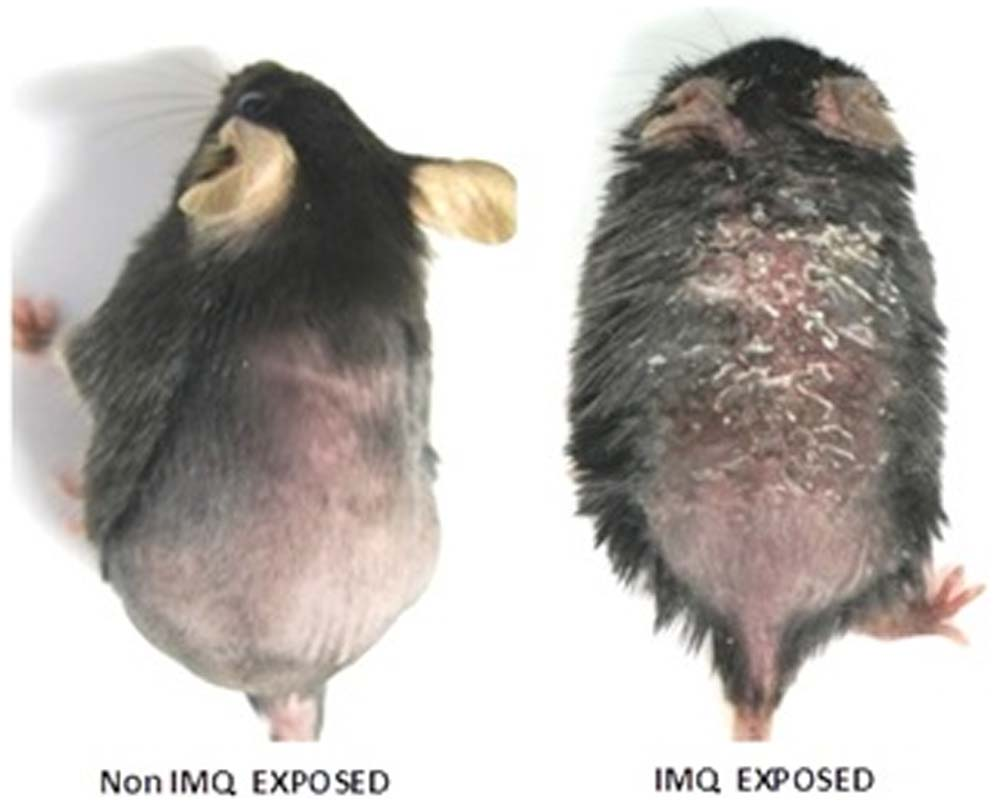
Mice without and with IMQ treatment.

The scaling of the dorsal skin of the mice, a phenomenon typical for psoriatic like skin lesions, was observed after topical application of IMQ (Fig. 13).

**Figure 14:**
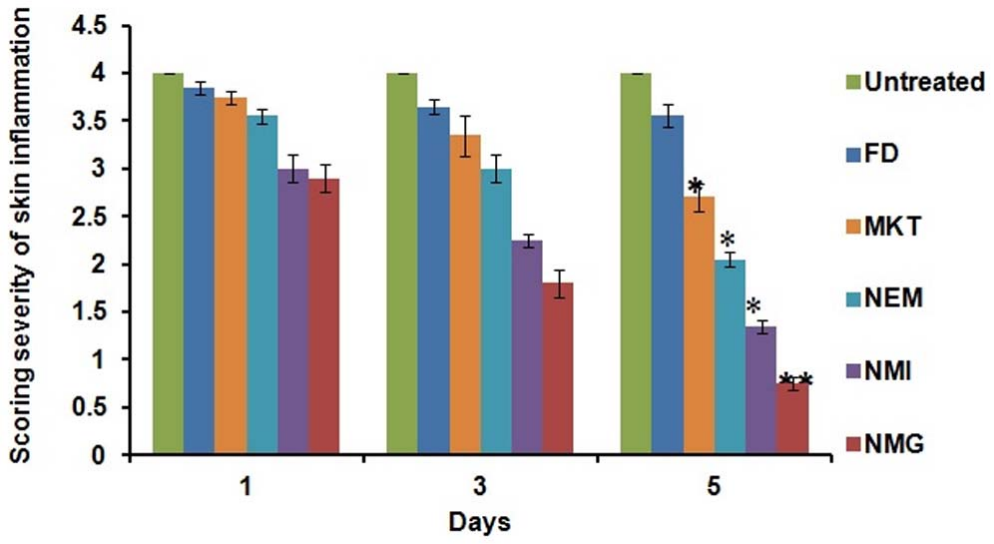
SSI (Scoring severity Index) values obtained from the scoring of the severity of skin inflammation in IMQ induced psoriatic like inflammation model of mice during the treatment with different formulations. Data represent mean±SD, n = 6, significant where *p<0.05 and **p<0.001 vs FD or positive control.

The PASI scores for all the groups were 4 at the end of 5th day of IMQ topical application. However, after 5 days of treatment, there was significant reduction in the inflammation induced by IMQ for NMG (p<0.01) and MKT (p<0.05). The PASI scores for FD, MKT, NEM, NMI and NMG formulation treated groups were decreased to 3.55±0.17, 2.70±0.15, 2.05±0.13, 1.35±0.10 and 0.75±0.07, respectively (Fig. 14).

**Figure 15:**
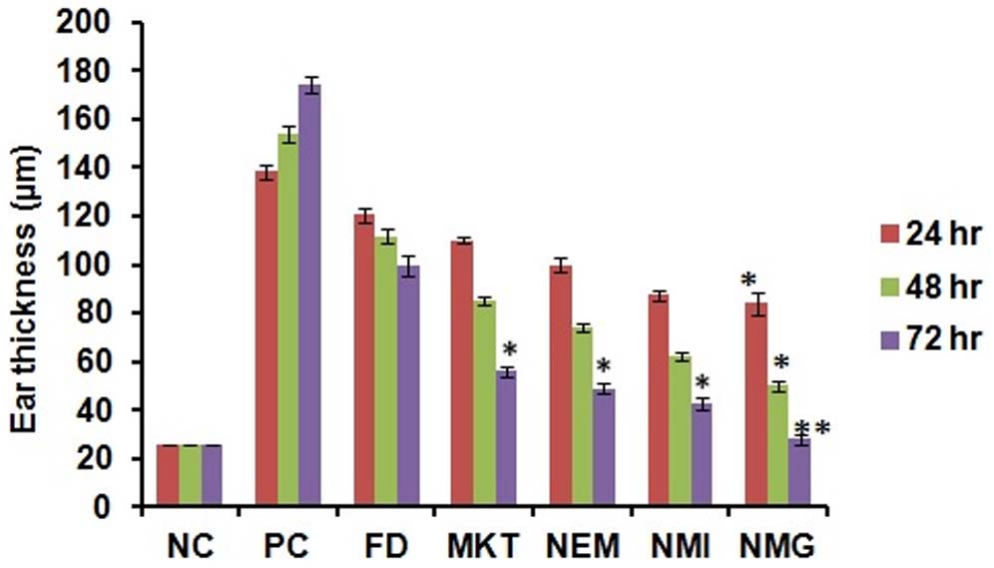
Difference in ear thickness of mice during treatment with different formulations. Data represent mean±SD, n = 6, significant where *p<0.05 and **p<0.001 vs FD or positive control.

Also, imiquimod caused a pronounced increase (26.00±3.02 to 174.00±16.27 µm) in the ear thickness of the mice by the third day of topical application. However, NMG significantly (p<0.01) reduced the ear swelling by 3.22 and 2.01-fold more than FD and MKT, respectively. After topical application of the different formulations for 5 consecutive days, the ear thickness was decreased to 89±21 µm, 56±15 µm, 48±13 µm, 42±10 µm and 27±6 µm for FD, MKT, NEM, NMI and NMG respectively (Fig. 15).

**Figure 16:**
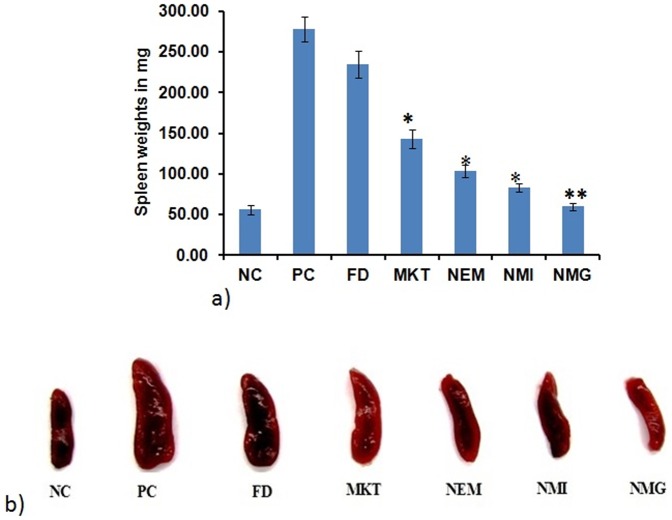
Illustration of the differences in spleen weights of the mice after treatment with different formulations for 5 days. Data represent mean±SD, n = 6, significant where *p<0.05 and **p<0.001 vs FD or positive control.

Further, topical IMQ increases spleen mass and alters its cellular composition. Increase in the percentage of macrophages and plasmacytoid dendritic cells are some unique features in IMQ treated mice. There was an increase in the spleen weight for IMQ only treated group which was significantly (p<0.01) reduced for NMG treated group at the end of the study, compared to FD and MKT. The spleen weights of FD, MKT, NEM, NMI and NMG were 1.18, 1.94, 2.69, 3.34 and 4.63-fold less than IMQ (positive control; PC). The average spleen weights for the different treatment groups, NC, PC, FD, MKT, NEM, NMI and NMG were 56.41±5.66 mg, 278.37±15.56 mg, 235.09±16.97 mg, 143.22±11.31 mg, 103.51±7.78 mg, 83.52±4.95 mg and 60.25±4.24 mg respectively (Fig. 16).

### Histology

**Figure 17:**
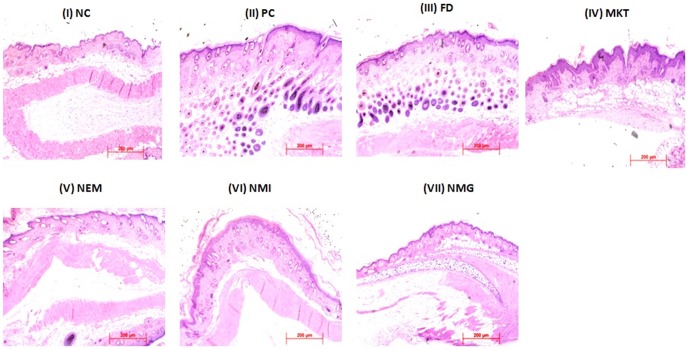
H&E histological staining showing altered keratinocyte proliferation and differentiation with IMQ exposure. C57/BL mice were exposed to the IMQ suspension for 5 days followed by treatment. i) Negative control (NC) ii) Positive Control (PC) iii) Free Drug (FD) iv) Marketed gel (MKT) v) Nano emulsion (NEM) vi) Nano micelle (NMI) vii) Nano miemgel (NMG). Data represent mean±SD, n = 6, significant where *p<0.05.

Analysis of H&E stained sections of IMQ-only treated skin (Positive control; PC) showed increased epidermal thickening with elongation of epidermal rete ridges, disturbed epidermal differentiation and infiltration of leukocytes into both the dermis and epidermis (Fig. 17). However, NMG showed minimum epidermal thickening and extension of the rete ridges with intact SC that was comparable to the negative control (no IMQ treatment, no drug treatment) but much less than the FD, NEM and NMI treatment groups. The general pattern of skin inflammation observed after the topical treatment of the inflamed dorsal skin was NC<NMG <NMI<NEM<<MKT<<FD<PC.

### Immunohistochemistry (IHC)

**Figure 18:**
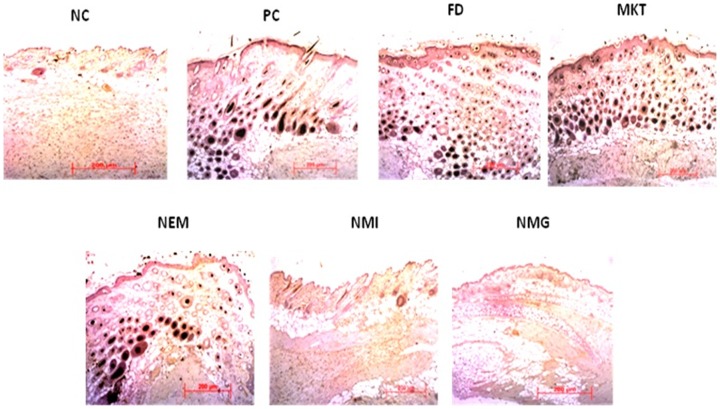
Immunohistochemistry IL-23 for i) Negative control (NC) ii) Positive Control (PC) iii) Free Drug (FD) iv) Marketed gel (MKT) v) Nano emulsion (NEM) vi) Nano micelle (NMI) vii) Nano miemgel(NMG). Data represent mean±SD, n = 6, significant where *p<0.05.

There was pronouncedly less brown staining for the IL 23 protein for NMG and NMI treated groups compared to the MKT, NEM and FD, which showed extensive brown staining that was comparable to the positive control (PC; IMQ only treatment). The general pattern of brown staining for IL-23 observed was NC<NMG<NMI<<MKT<NEM<<FD<PC (Fig. 18).

### Permeation through psoriatic like inflamed mice skin

**Figure 19:**
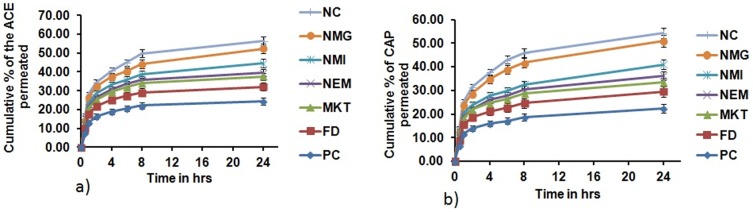
Permeation of a) Aceclofenac (ACE) & b) Capsaicin (CAP) through psoriatic like inflamed skin treated with different formulations. Cumulative % of ACE & CAP permeated after 24 h through psoriatic like inflamed skin from NMG was significantly more compared to PC, FD, MKT, NEM and NMI. Significant where p<0.05, Data represent mean±SD, n = 6, (*p<0.05 when NMG vs MKT, NEM, NMI; **p<0.001 when NMG vs PC, FD).

The objective of the permeation study through the inflamed psoriatic like skin portions of the mice treated with the different formulations was to find out if there was any significant difference in the permeation resulting from the topical treatment with the different formulations. Permeation of ACE and CAP through the psoriatic skin portions of mice treated with different formulations is depicted in Fig. 19. The amounts of ACE and CAP that permeated into the receptor compartment from NMG after 24 hr through the PC and NC treated psoriatic skin portions were found to be the highest and lowest, respectively, among all the results observed.

**Figure 20:**
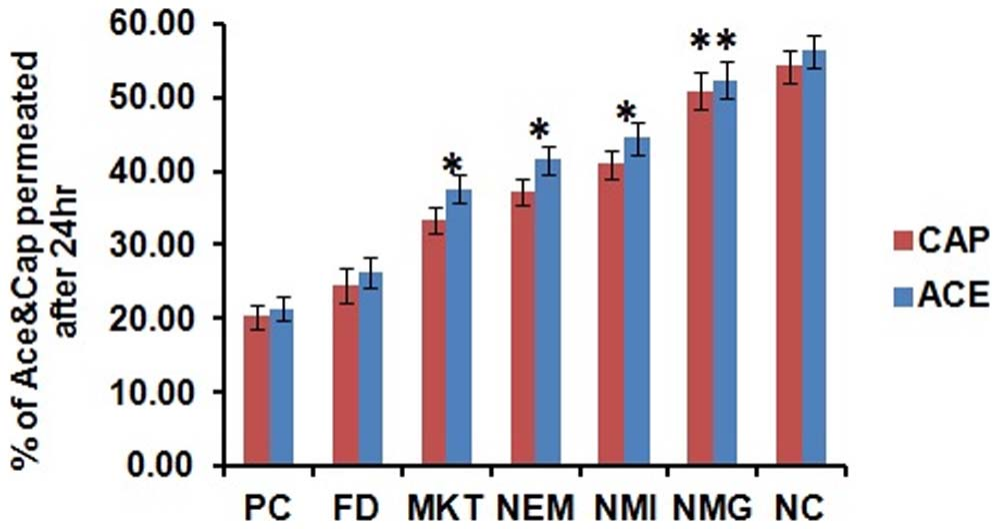
Total permeation of ACE & CAP after 24 h through psoriatic like inflamed mice skin treated with different formulations. Data represent mean±SD, n = 6, significant where *p<0.05 and **p<0.001 vs FD or positive control.

The amounts of ACE and CAP that permeated into the receptor compartment after 24 hr through the FD and NMG treated psoriatic skin portions were similar to the PC and NC, respectively (Fig. 20). NMG treated psoriatic skin portion showed significantly more permeation of ACE and CAP from NMG than FD (p<0.01) and MKT (P<0.05). The permeation was 2.0 and 2.08-fold more than FD and 1.39 and 1.52-fold more than MKT of ACE and CAP, respectively (Table 3).

**Table 3:** Percentage of drug permeated through the psoriatic-like inflamed skin from NMG after being treated with different formulations.

Formulation	Percentage of drug permeated through the psoriatic-like inflamed skin								
	PC	NC	FD	MKT	NEM	NMI	NMG	NMG/FD	NMG/MKT
ACE	21.33±1.65	56.28±2.25	26.17±2.05	37.58±1.88	41.59±1.98	44.48±2.12	52.38±2.52	2.00	1.39
CAP	20.29±1.68	54.23±2.17	24.48±2.36	33.43±1.67	37.27±1.81	40.92±1.95	50.88±2.44	2.08	1.52

Data expressed as mean±SD (n = 6), significant where *p<0.05.

### Combination Index

The combination Index (CI) values for the different parameters like cumulative percent of drug permeated into the receptor compartment through the dermatomed human skin, amount of drug retained in the different layers of the skin and the inflamed ear thickness of mice after induction by IMQ was in the range of 0.71 to 0.76.

## Discussion

It is desirable to deliver the therapeutic agent for treating inflammatory skin diseases into the deeper epidermal and dermal layers to achieve maximum therapeutic success [27]. Many researchers have utilized nanoparticulate systems to enhance the skin permeation and deposition of the payloads they carry. However, permeation studies have demonstrated that nanoparticles do not cross the SC but possibly lodge in the SC layers and release the encapsulated drug in a controlled manner into the upper epidermis; the released drug then passively diffuses into the skin layers below [28]. Although, recent investigations have demonstrated that nanoparticles can transport the loaded agents into the skin layers through hair follicles, the total amount reaching the dermal site is very limited [28].

In the present study, we prepared a novel drug delivery system NMG by combining two different drug delivery systems (NEM and NMI) and incorporating them into a carbopol gel. The performance of the combination was compared with the individual drug delivery systems both in *in vitro* and *in vivo* and we observed that there was significantly (P<0.05) enhanced permeation of both ACE and CAP into the deeper skin layers delivered by NMG than the FD, NMI, NEM and MKT respectively.

Carbopol was selected as the gelling agent for NMG because it forms a homogenous dispersion due to its high water solubility and ability to form a transparent gel after the addition of an alkaline reagent [29]. The effect of the different amounts of carbopol on the viscosity profile and particle size of NMG was investigated because minimum particle size was desired for the viscous gel. The 2% w/v carbopol gel showed greater viscosity and particle size compared to the 1% carbopol gel possibly because there was increased cross-linking of the polymeric network; hence it was selected for the rheological investigations of NEM, NMI and NMG because the spreadability and contact time of a topical gel on the skin surface is directly related to its rheological behavior [30].

Also, the texture analysis showed that there was higher firmness (294.61 g) and consistency (1402.70) but lesser cohesiveness (−41.12) of the MKT gel and this can be attributed to the nature of the ingredients used to prepare the gel. Further, the lack of sufficient water content possibly caused the gel to harden and this contributed to the high resistance observed during the entry of probe into the gel. This resulted in the force 1 & area of force vs time (−158.45) between line one and two of TA curve being higher. The texture parameters of the NMG contrarily were close to an average of the parameters for NEM and NMI because NMG is a combination of the two. The substantial firmness and consistency properties observed for NEM were possibly due to the higher oil content of the nano formulation. However, there was a higher cohesiveness observed generally in the nano formulations than the MKT due to the addition of 0.5% Pluronic F-125 to the 2% carbopol gel. This possibly resulted in the enhanced permeation of the drugs observed, through the formation of a thin film on the skin surface and the production of a good occlusive effect.

Further, the nano formulations stored at 2–4°C and room temperatures showed excellent stability without significant increase in the particle size and drug content during the entire study period, which may be attributed to the hydration effect of carbopol. However, a gradual increase in both the particle size and distribution was observed at accelerated storage conditions, possibly because there was an increased loss of water at the higher storage temperatures, which caused the aggregation of the particles and the destabilization of the system.

Importantly, the *in vitro* drug release from NEM, NMI and NMG followed Korsmeyer Peppas kinetics demonstrating that the drug was released in a controlled manner through a combination of both diffusion and erosion mechanisms. Also, the controlled drug release from NEM, NMI and NMG may be due to the longer diffusion pathway caused by the entrapment of the drug in nano formulation [31]. The skin permeation studies on the other hand showed a significant (p<0.05) increase in ACE and CAP penetration into the receiver compartment as well as drug retention in the deeper skin layers generally for the nano formulations (NEM, NMI and NMG) compared to the FD and MKT. The analysis also showed that NMG formed drug deposits in the skin layers, which resulted in the sustained release of the drug over 24 hr with a concentration that was about 3-fold higher than MKT, although MKT also induced sustained drug release, possibly due to the inherent strong protein binding nature of both ACE and CAP. The penetration of NMG through the skin can be due to the usage of both of the routes that are utilized by NEM and NMI; hence, NMG permeated the skin by both paracellular and passive diffusion across the skin cells to deliver its payloads into the deeper layers of the skin.

As every drug delivery system is unique, its rate, extent and mechanism of absorption depend on the size, charge and composition of the drug delivery system. So, when a combination of completely different drug delivery systems (NEM+NMI) was utilized for the delivery of a drug, the absorption of the combined system (NMG) was found to be better than either of the individual drug delivery systems due to the utilization of the maximum possible paths of absorption available for that particular drug.

In this work, micelles were prepared using vitamin E TPGS and it was established fact that surfactants disturb the arrangement of cells temporarily and thereby enhance the absorption of poorly soluble drug molecules which otherwise called para-cellular absorption whereas emulsion was prepared using olive oil and because of their lipophilicity and nano size they were assumed to be taking the advantage of trans-cellular route.

This was also evident from the observation of more intense green fluorescence signal in deeper skin layers from FITC loaded NMG clearly demonstrated its superior skin penetration ability over NEM and NMI thereby strengthened the hypothesis of the multi absorption mechanism with combination of two different drug delivery systems. These results were also comparable and well correlated with the in vitro skin permeation studies conducted through dermatomed human skin.

The microdermal dialysis study of NMG vs MKT demonstrated that ACE and CAP could be safely delivered into the skin of hairless rat in an enhanced approach with NMG more than the MKT. There were no signs of irritation observed even after 24 hrs at the site of application with both NMG and MKT. The higher C_max_ values of NMG signify its better permeation effect possibly because of the presence of permeation enhancers, the nano size and the unique combination of the two drug delivery systems (NMI and NEM). The increased t_max_ and elimination half life of NMG signifies the sustained release of ACE and CAP, which could be because of the efficient entrapment of both drugs in the nanocarrier. It should be noted that both gels (NMG and MKT) were left on the surface of the hairless mice skin for 24 hours.

Aceclofenac, the glycolic acid ester of diclofenac inhibits the cyclo-oxygenase enzyme (COX) involved in the production of prostaglandins that cause pain, swelling and inflammation [32]. Capsaicin on the other hand, selectively binds to a protein known as TRPV1 that resides on the membranes of pain and heat-sensing neurons. Capsaicin helps in the treatment of inflammation by depleting the presynaptic substance P, which is one of the body's neurotransmitters for pain and heat [33].

During skin inflammatory disorders like psoriasis, various cytokines, chemokines, eicosanoids and substance P has been reported to play vital roles in the regulation of the inflammatory process. Psoriasis is an acute inflammation, characterized by classical symptoms such as heat, redness, scaling, skin thickness, swelling and pain. Further, IMQ has been reported to induce skin inflammation in mice that exhibits the classical phenotypic and histological features of human psoriasis including the up regulation of cytokines like IL-23 and IL-17 [34]. Therefore the efficacy of NMG was investigated in an IMQ induced psoriatic-like plaque model developed in mice. Note worthily, the H&E staining demonstrated all the classical symptoms mentioned above for the clinical psoriasis presentations, for the positive control (IMQ only treatment). Further, the extent of epidermal thickening, elongation of epidermal ridges and brown staining for IL-23 protein was much less for the nano formulations (NMG, NMI and NEM) and MKT than FD. The histological characteristics of skin treated with FD were comparable to the positive control with extensive brown staining of IL-23, hence demonstrating the inefficiency of the formulation to adequately deliver therapeutics amounts of ACE and CAP at the epidermal and upper dermal regions for anti-inflammatory actions. Also, all the nano formulations showed less epidermal thickening and acanthosis than MKT possibly due to the improved delivery of drug into the deeper skin layers by its multi absorption mechanisms. However, among the nano formulations, the NMG exhibited markedly reduced inflammation and histological features that were comparable to the negative control (no IMQ and no drug treatment) and much less than the NMI and NEM (p<0.05). This suggests that because the NMG is a combination of NMI and NEM, it possesses improved topical delivery properties that make it superior to the two formulations. This observation was also notable in the significant (p<0.01) decrease in spleen weights caused by the 5-day treatment with NMG following IMQ-induced inflammation, compared to MKT and all the other treatment groups. There was no significant difference in the spleen weights between the NC (Negative control) and NMG and this is suggestive of the fact that NMG was capable of delivering therapeutic amounts of both ACE and CAP into the epidermal and dermal layers of the skin to reduce the inflammation induced by IMQ treatment.

Permeation through psoriatic inflamed skin is expected to be less because of the development of plaque, scaling, epidermal alterations, epidermal thickening and elongation of epidermal ridges, which create barriers to the penetration of drugs through the skin. This hence may account for the decreased transport of drugs into the skin by topical drug delivery systems during psoriasis treatment. This observation has been demonstrated by Shinji Oshima et al. [35] who have shown in their studies that flurbiprofen plasma concentration following the topical application of ZEPOLAS was suppressed in rats presenting with inflamed skin. They also observed that there was decreased permeation of the drug into the receptor compartment through the inflamed rat skin compared to the normal skin in *in vitro* and microdialysis experiments. The results from the present study were hence in agreement with their report because after 24 hr *in vitro* permeation studies with NMG through the differently treated inflamed rat skin (5 day treatment with NMG, NMI, NEM, FD and MKT following IMQ-induced skin inflammation), there was a significant (p<0.01) increase in penetration of ACE and CAP into the receptor compartment through NMG-post treated inflamed skin compared to MKT and FD. This observation further illustrates the efficiency of NMG to deliver ACE and CAP into the deep layers of the skin for the complete inhibition of inflammation of the skin to a level that was comparable to the negative control (no IMQ treatment).

To ascertain whether the combination of NEM and NMI (NMG) was additive or synergistic, the combination index value was calculated for all the *in vitro* and *in vivo* tests conducted. The combination Index (CI) values calculated for the different parameters were in the range of 0.71 to 0.76 indicating the moderate synergism of NMG. The reason for this synergism could be the utilization of all the possible absorption pathways by NMG, which results in the enhanced permeation and retention of drug in skin and thereby produces a superior therapeutic effect than either NEM or NMI.

## Conclusion

Our studies demonstrate that the NMG comprising NEM and NMI enhanced the skin permeation of aceclofenac and capsaicin by translocating the nanoparticles across the deeper skin layers by improving the skin contact time, hydrating the skin and by forming a thin layer on the skin surface (occlusive effect). Thus, the increase in skin permeation of aceclofenac and capsaicin was further responsible for the improved therapeutic response of the psoriatic plaque like model to the treatment of ACE and CAP-containing NMG suggesting the potential of this combination therapy to treat psoriasis. Further, the data of microdialysis demonstrates that the NMG was superior to the MKT. This approach can also be extrapolated to the delivery of therapeutics for treating other skin diseases like fungal, bacterial, viral infections and skin cancers like melanoma. However, more experiments are necessary to prove the absorption mechanism of NMG to find out the exact factors contributing to its synergistic effect over NMI and NEM. Future studies will be directed to the use of more potent drugs with the NMG approach for the treatment of various skin disorders like psoriasis, allergic contact dermatitis, acne etc.
