# Correction: Nanomiemgel—A Novel Drug Delivery System for Topical Application—In Vitro and In Vivo Evaluation

**DOI:** 10.1371/journal.pone.0120951

**Published:** 2015-03-20

**Authors:** The PLOS ONE Staff

The following information is missing from the Funding section: Dr. Jaganmohan Somagoni received support from the NIH NCRR RCMI program, G12RR 03020.

The complete, correct Funding section is as follows: Dr. Jaganmohan Somagoni received support from the NIH NCRR RCMI program, G12RR 03020. The funders had no role in study design, data collection and analysis, decision to publish, or preparation of the manuscript.
